# Spatiotemporal representation of cardiac vectorcardiogram (VCG) signals

**DOI:** 10.1186/1475-925X-11-16

**Published:** 2012-03-30

**Authors:** Hui Yang, Satish TS Bukkapatnam, Ranga Komanduri

**Affiliations:** 1 Department of Industrial & Management System Engineering, University of South Florida, Tampa, FL, USA; 2 Department of Industrial Engineering & Management, Oklahoma State University, Stillwater, OK, USA; 3 Mechanical & Aerospace Engineering, Oklahoma State University, Stillwater, OK, USA

**Keywords:** Vectorcardiogram (VCG), Electrocardiogram (ECG), Spatiotemporal representation, Color-coding scheme

## Abstract

**Background:**

Vectorcardiogram (VCG) signals monitor both spatial and temporal cardiac electrical activities along three orthogonal planes of the body. However, the absence of spatiotemporal resolution in conventional VCG representations is a major impediment for medical interpretation and clinical usage of VCG. This is especially so because time-domain features of 12-lead ECG, instead of both s*patial* and *temporal* characteristics of VCG, are widely used for the automatic assessment of cardiac pathological patterns.

**Materials and methods:**

We present a novel representation approach that captures critical spatiotemporal heart dynamics by displaying the real time motion of VCG cardiac vectors in a 3D space. Such a dynamic display can also be realized with only one lead ECG signal (e.g., ambulatory ECG) through an alternative lag-reconstructed ECG representation from nonlinear dynamics principles. Furthermore, the trajectories are color coded with additional dynamical properties of space-time VCG signals, e.g., the curvature, speed, octant and phase angles to enhance the information visibility.

**Results:**

In this investigation, spatiotemporal VCG signal representation is used to characterize various spatiotemporal pathological patterns for healthy control (HC), myocardial infarction (MI), atrial fibrillation (AF) and bundle branch block (BBB). The proposed color coding scheme revealed that the spatial locations of the peak of T waves are in the Octant 6 for the majority (i.e., 74 out of 80) of healthy recordings in the PhysioNet PTB database. In contrast, the peak of T waves from 31.79% (117/368) of MI subjects are found to remain in Octant 6 and the rest (68.21%) spread over all other octants. The spatiotemporal VCG signal representation is shown to capture the same important heart characteristics as the 12-lead ECG plots and more.

**Conclusions:**

Spatiotemporal VCG signal representation is shown to facilitate the characterization of space-time cardiac pathological patterns and enhance the automatic assessment of cardiovascular diseases.

## Background

The electrocardiogram (ECG) signals are recorded on the body surface to track the continuous dynamic details of cardiac functioning. Such valuable real-time information is usually unavailable in static and discrete clinical laboratory tests, for e.g., computer imaging, chest x-ray, and blood enzyme test. Even if routine laboratory examinations are performed multiple times per day, discontinuity often fails to prevent the lethal consequences from acute cardiac disorders. There is an increasing awareness that real-time ECG monitoring is an essential tool for the early identification of cardiac pathological patterns because it tracks cardiac dynamic behaviors as opposed to static screenshots.

However, one lead ECG signals only capture one perspective temporal view of the space-time excitation and propagation of cardiac electrical activities. Multiple lead ECG systems, for e.g., 12-lead ECG and 3-lead vectorcardiogram (VCG), are designed to capture the multi-directional view of space-time cardiac electrical activities. The 12-lead ECG is more commonly used than the 3-lead VCG because medical doctors are accustomed to using them in clinical applications. It has thus proven its value, time tested, and considered as the Gold Standard. However, much of that information is redundant and even in that, only a small fraction of the data is used in the analysis by the physicians based on experience, expertise, and oftentimes on the memorization of ECG signals for different cardiac disorders. This is a difficult task and cardiologists are constantly looking for more accurate and effective alternatives. In addition, when it comes to the automated computer analysis of heart electrical activity, 12-lead ECG signals have higher dimensionality than 3-lead VCG and can potentially introduce the “curse of dimensionality” problem. VCG surmounts not only the information loss from only one or two ECG signals but also the dimensionality problems induced by the 12-lead ECG signals. In this paper, we propose a new dynamic display of VCG to characterize both spatial and temporal patterns of cardiac electrical activities as an alternative for the conventional 12-lead ECG.

**Figure 1:**
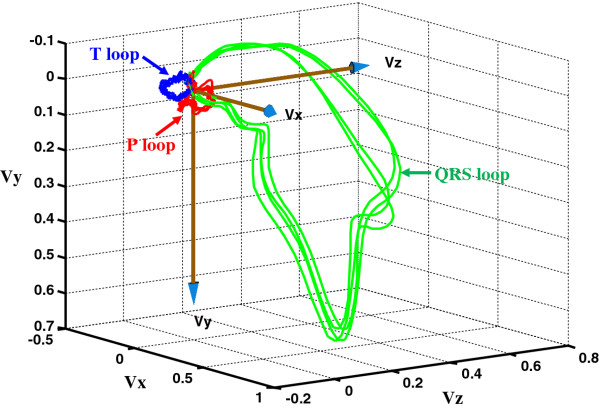
**A representative VCG plot showing vector loops for P, QRS, and T wave activities.** The largest green QRS loop manifests the ventricular depolarization activities. Red P wave is the atrial depolarization after the SA node excitation. The ventricular repolarization is shown as blue T wave loop.

VCG signals monitor the cardiac electrical activities along three orthogonal planes of the body, namely, frontal, transverse, and sagittal. As shown in Figure 1, the VCG vector loops contain 3 dimensional recurring, near-periodic patterns of heart dynamics [1]. Each heart cycle consists of three loops corresponding to P, QRS, and T wave activities. The VCG signals are traditionally projected onto different planes (X-Y plane, X-Z plane and Y-Z plane) to capture the time correlations, or plotted as a static attractor in a 3D space that provides the topological relationships. The absence of combined spatial and temporal information in the VCG representations reported earlier expects the interpreters to have knowledge on both spatial and temporal patterns of cardiac events.

With rapid advancements in information technology and the availability of computing hardware at reasonable costs, representation of 3D VCG loops are not constrained by computational resources anymore and this has generated renewed interest in VCG since the 1990’s. Dower and his colleagues [2-4] conducted pioneering research based on Frank’s tank torso model studies and introduced a linear transformation matrix to derive 12-lead ECG signals from 3-lead VCG signals. Such a transform was shown to preserve clinically useful information pertinent to heart dynamics. It may be noted that the 3 orthogonal leads, e.g. the corrected Frank leads, contain the all necessary information but very often the signal amplitudes are low and superimposed by noise. In such cases the redundancy is extremely useful. Besides, the orthogonal leads are acquired via uncomfortable electrode positions (i.e., on the patient back). Therefore, the inverse transform is often used to derive the 3-lead VCG from the 12-lead ECG [5]. Some even consider Dower transformation matrix as “generalized” or “universal” transformation matrix [2-4,6] although there is a need to develop different transformation matrices for healthy subjects and patients with cardiac disorders [7].

For a given cluster of subjects, e.g., healthy control subjects in certain age and gender group, the transformation studies show statistical equivalence between the 12-lead ECG and 3-lead VCG signals [2-4,6,7]. However, the spatial information of cardiac vectors is hardly recognized in the conventional 12-lead electrocardiogram (ECG) plots, and temporal aspects are less discernible in the traditional visualization of 3D static VCG vector trajectory. In addition, it is difficult for human beings to visually project a spatial VCG vector into any specified angle in the 12-lead measurement system, which is the traditional way for the interpretation of ECG signals. It may be noted that the 12-lead system present the necessary information in more than 90% of the cases. The VCG leads are useful specifically for deriving details that are pertinent to myocardial infarctions. Therefore, the physicians very often interpret in parallel the 12 leads and the VCG loops.

It is well known that the 12-lead ECG is heavily depended on temporal information, for e.g., intervals and durations, but such temporal aspects are absent in the static VCG representation. Hence, this poses extra difficulties to closely relate the 12-lead ECG characteristics (e.g., QT interval, ST elevation) to the patterns of 3-lead Frank X, Y, Z VCG signals. Although the medical interpretation and clinical usage of VCG have been investigated by many researchers [8-13], the dynamic representation of both spatial and temporal aspects in VCG signals are rarely, if any, to be found. Most previous investigations studied the pathological patterns in the static VCG signals and exemplified the advantages of VCG in the automatic assessment of certain cardiovascular disorders. Spatial VCG signals were shown to not only facilitate in the basic understanding of the electrical phenomena associated with the heart but also disclose pathological characteristics unknown, or not feasible, from the ECG signals [14,15]. This was experimentally validated for different cardiovascular disorders including the right and left ventricular hypertrophy, singly or combined, intraventricular block, and myocardial infarction [14,15]. This paper presents a dynamic VCG signal representation approach to capture critical spatiotemporal heart dynamics by displaying the real time motion of 3-dimensional VCG cardiac vectors on a computer screen.

## Materials and methods

In this investigation, we have used 3-lead VCG signals gathered from the PTB database available in the PhysioNet [16,17]. Each of the recordings in the PTB database contains 15 simultaneously recorded signals, namely, the conventional 12-lead ECGs and the three orthogonal Frank XYZ lead VCG signals digitized at 1 kHz, with a 16 bit resolution over a range of ±16.384 mV. The database used consists of 80 HC recordings acquired from 54 healthy volunteers and 368 MI recordings from 148 patients. The recordings were collected at the Department of Cardiology of University Clinic Benjamin Franklin in Berlin, Germany.

### Description of the method

**Figure 2:**
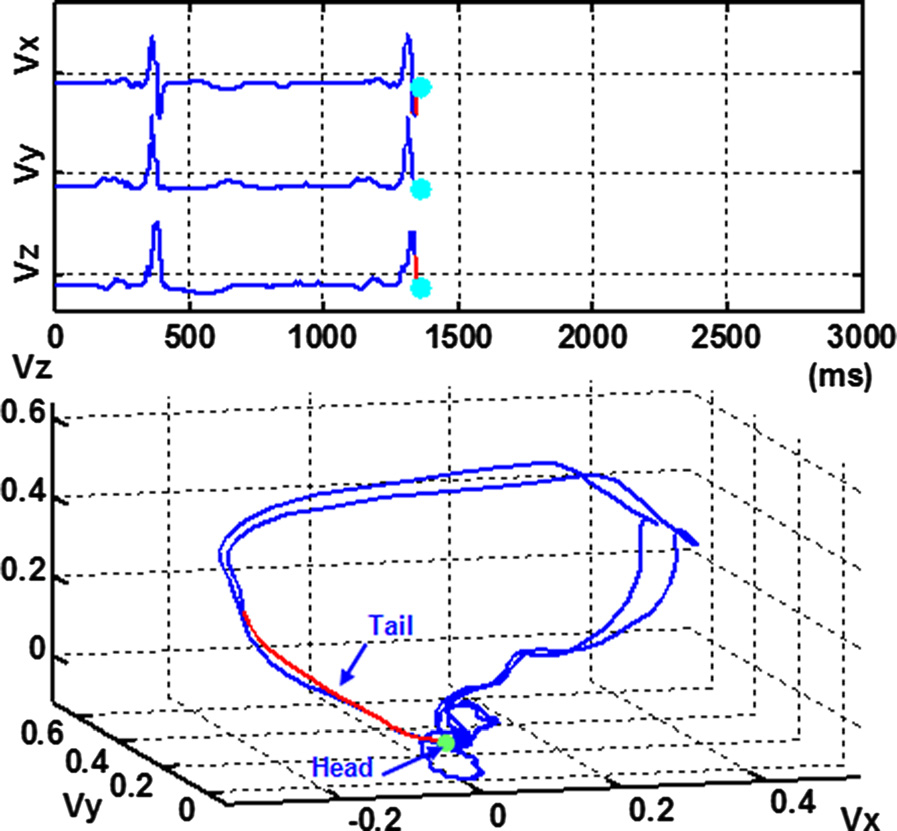
**Spatiotemporal VCG representation of a patient with myocardial infarction, anterior.** The top plot displays VCG signals in three vector components as a function of time, and the bottom part shows the real time cardiac vector movement in the 3D space. Head (green) gives the current position of cardiac vector. Tail (red) indicates the direction and rotation of cardiac vector movements (see animation video in Additional file 1).

As shown in Figure 2, the VCG vector loops contain 3 dimensional recurring, near-periodic patterns of heart dynamics, which can be visualized in the X-, Y- and Z- space domain with time entering implicitly. The dynamic VCG signal representation provides an easier way to understand, interpret, and use space-time information of cardiac electrical activities. This approach includes presenting the real time motion of cardiac vectors in 3D space and color coding of cardiac vector movements with some dynamical properties, for e.g., curvature, spatial octant [18], velocity, and phase angle. For one dimensional heart monitoring signals, an alternative lag-reconstructed representation from nonlinear dynamics principles is provided. In addition, the Poincaré sectioning of the 3D VCG vector loops extracts the homogeneous ECG ensembles for selective averaging. The cycle-to-cycle self-similarities and variations provide valuable information about the majority clustering of ECG morphology and heart rate variability. The proposed dynamic representation of cardiac vectors can be rotated freely on the computer monitor screen for the spatiotemporal analysis.

A. Spatiotemporal VCG signal representationIn the Frank XYZ lead system, VCG is represented as three orthogonal scalar measurements with respect to time as given in Eq.1. The dynamic VCG signal representation embeds the cardiac vector, composed of three scalar measurements, in real time. As shown in Figure 2, three scalar x, y, z components are plotted in the top and the simultaneous 3D movement of cardiac vectors in the bottom.

(1) $$\left\{ \begin{array}{c} v_{x}=f\left(t\right)\\ v_{y}=g\left(t\right)\\ v_{s}=h\left(t\right) \end{array}\right.$$

Therefore, this explicitly real time spatiotemporal VCG representation makes it easier to integrate with prior knowledge and experiences of time-based ECG. As shown in Figure 2, this representation consists of three components, namely, head (green), body (red), and tail (blue). Head gives the current position of the cardiac vector. Body records a short history of the cardiac vector movements which clearly indicates where the current vector is from. It avoids the confusion regarding which group of heart activity the current cardiac vector belongs to as they usually intersect at the isoelectric points. The tail provides full history pertinent to the complete topological shape of VCG state space. By following the cardiac vector movement with respect to time, the P, QRS, and T waves will be easily located in the VCG state space (see Figure 2).

**Figure 3:**
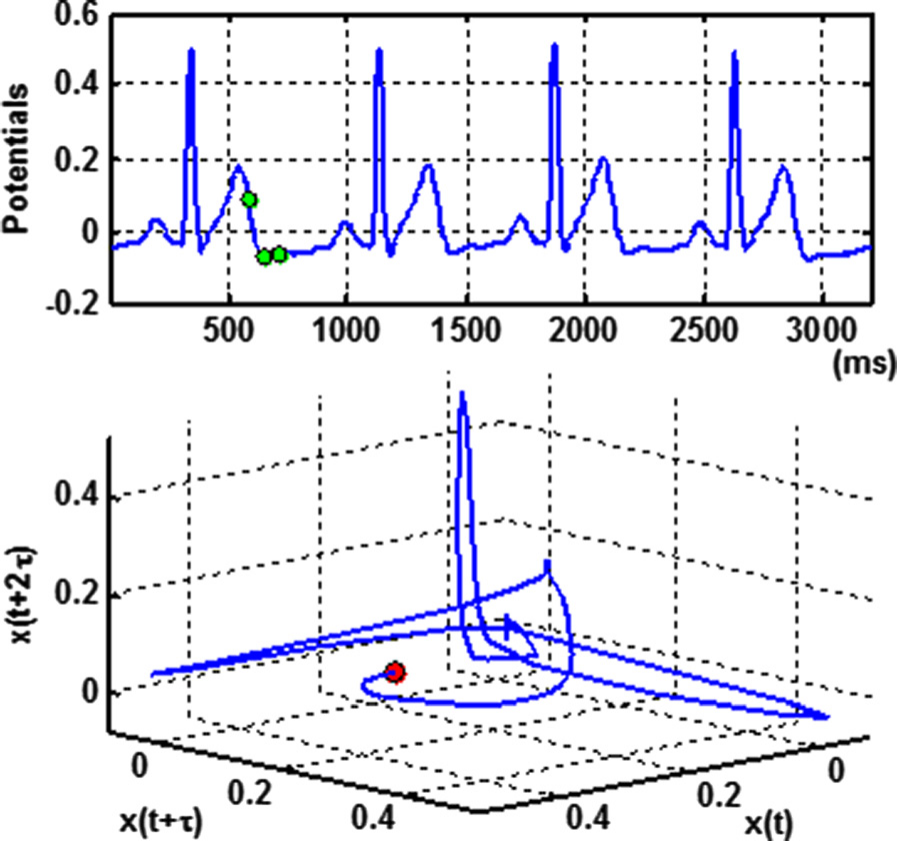
**Lag-reconstructed dynamic ECG representation for a normal subject.** Time delay coordinates of the original one dimensional ECG time series are used to embed the 3D manifold (see animation video in Additional file 2).

B. Lag-reconstructed spatiotemporal ECG representationIn some real-world cases, for e.g., ambulatory ECG, only a single channel ECG is available in lieu of the complete measurements of three cardiac vector components. Takens embedding theorem [19] states that the individual measurements contain adequate information to reconstruct pseudo state space because of the high couplings in real-world complex systems. Although a single channel ECG is hardly presented in the space-time form as cardiac vectors, a pseudo state space (attractor) can be reconstructed from delayed coordinates of the single measurement *y*(*t*) as

(2) $$\underset{￣}{v}\left(t_{i}\right)=\left[y\left({\text{t}}_{i}\right),y\left({\text{t}}_{i}+\tau \right)\text{,}\;y\left({\text{t}}_{i}+2\tau \right)\right]$$

where τ is the time delay. The optimal time delay τ is selected to minimize mutual information function *M*(*τ*), defined as

(3) $$M\left(\tau \right)=\int_{t}p\left(t,t+\tau \right)\log\frac{p\left(t,t+\tau \right)}{p\left(t\right)p\left(t+\tau \right)}dt$$

where *p*(t,τ) is the joint density function, and *p*(*t*) and *p*(*t +* τ) are marginal density functions of *y*(*t*) and *y*(*t +* τ), respectively [19]. As shown in Figure 3, the animation of lag-reconstructed ECG attractor will provide space-time information when the multi-dimensional ECG signals are not readily available. It may be noted that the lag-reconstruction approach was widely used in physics to explore the nonlinear dynamics underlying the ECG signals [20-22]. Examples of the nonlinear dynamical quantifiers may include Lyapunov exponents, recurrence statistics, and correlation dimension, which describes the characteristics of the signals. This paper presents the lag reconstruction approach to facilitate the real-time spatiotemporal representation when 3-lead VCG, i.e., actual state space, is not available.

C. Color coding of spatiotemporal VCG signal representationThe hardware apparatus for recording “color vectorcardiogram” was previously designed using camera, filter, lens and oscilloscope to facilitate the understanding and increase the diagnostic scope of vectorcardiography [23]. This paper presents a software color-coding scheme to incorporate additional dynamical attributes of spatiotemporal VCG signals as opposed to use hardware devices. It will not only overcome the color resolution drawbacks from the limitations of hardware but also reduce the economic costs of VCG monitoring systems. The dynamical properties for the coloring of VCG vector loops may include, but not limited to, speed (***v***′(*t*) = Δ***v****/*Δ*t*, and Δ***v*** *= ||****v***(*t*)*-****v***(*t+*Δ*t*)*||*), phase angles, octant numbers, and curvature. The phase angles provide similar information as octant numbers, but in fine-grained scales. The phase angles of cardiac vectors can be determined as the following equation:

(4) $$\cos\theta =\frac{v_{x}}{\sqrt{v_{x}^{2}+v_{y}^{2}+v_{z}^{2}}},\cos a=\frac{v_{x}}{\sqrt{v_{x}^{2}+v_{y}^{2}+v_{z}^{2}}},\text{and cos}\;\beta =\frac{v_{x}}{\sqrt{v_{x}^{2}+v_{y}^{2}+v_{z}^{2}}}$$

The eight octants delimit the cardiac vectors in the scale of 90^0^. The VCG trajectory in the 3D space is color coded with eight different colors with respect to the octant numbers. As can be noted from Table 1, a binary number with three bits (binary digits) is used to designate each octant. Binary code 0 is used to represent the negative directions in X-, Y-, and Z- axes and 1 for the positive directions [18]. For example, if the octant lies in the (−, +, −) XYZ directions, binary coding for this will be (010) and the resulting octant number is 2 (binary coding: 0 × 2^2^ + 1 × 2^1^ + 0 × 2^0^ = 2).If the VCG is denoted as three orthogonal scalar measurements with respect to time as $v\left(t\right)=<v_{x},v_{y},v_{z}>=<f\left(t\right),g\left(t\right),h\left(t\right)>,a\leq t\leq b$ and the cardiac trajectory is traversed as *t* increases from *a* to *b*, then it can be shown that the length of such a space curve is $L=\int_{a}^{b}\left|v^{\prime }\left(t\right)\right|dt=\int_{a}^{b}\sqrt{{\left[f^{\prime }\left(t\right)\right]}^{2}+{\left[g^{\prime }\left(t\right)\right]}^{2}+{\left[h^{\prime }\left(t\right)\right]}^{2}}dt$. The arc length *s* as a function of *t* is $s\left(t\right)=\int_{a}^{t}\left|v^{\prime }\left(u\right)\right|du=\int_{a}^{t}\sqrt{{\left[f^{\prime }\left(u\right)\right]}^{2}+{\left[g^{\prime }\left(u\right)\right]}^{2}+{\left[h^{\prime }\left(u\right)\right]}^{2}}du$. The unit tangent vector giving the speed direction at a particular point is calculated by the following formula: $T\left(t\right)=v^{\prime }\left(t\right)/|v^{\prime }\left(t\right)|$. The curvature, at a given point, of the cardiac VCG trajectory is defined as the rate of change of the unit tangent vector with respect to arc length. In other words, the curvature at a point indicates how fast the trajectory is bending at that point. Therefore, the curvature $\kappa \left(t\right)$ of 3-lead VCG trajectory is calculated as follows:

**Table 1:** VCG octant positions and color coding

**Octant**	**X**	**Y**	**Z**	**Binary code**	**Location (X, Y, Z)**	**Color code**
0	−	−	−	(000)	Right -superior-anterior	Black
1	−	−	+	(001)	Right -superior-posterior	Blue
2	−	+	−	(010)	Right -inferior-anterior	Gray
3	−	+	+	(011)	Right -inferior-posterior	Cyan
4	+	−	−	(100)	left-anterior-superior	Magenta
5	+	−	+	(101)	left -superior-posterior	Green
6	+	+	−	(110)	left -inferior-anterior	Yellow
7	+	+	+	(111)	left -inferior-posterior	Red

(5) $$\kappa \left(t\right)=\left|\frac{dT}{ds}\right|=\left|\frac{dT}{dt}\right|*\left|\frac{dt}{ds}\right|=\frac{\left|T^{\prime }\left(t\right)\right|}{\left|v^{\prime }\left(t\right)\right|}=\frac{\left|v^{\prime }\left(t\right)\times v^{\prime \prime }\left(t\right)\right|}{{\left|v^{\prime }\left(t\right)\right|}^{3}}$$

**Figure 4:**
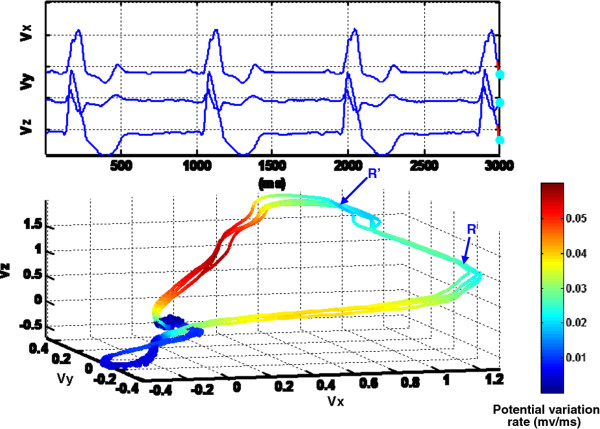
**Dynamic VCG representation plot of a patient with bundle branch block.** The 3D VCG trajectory is color coded with the potential variation rate listed in the right side. Two curly R peaks designated as “M waves” are shown in the QRS loop. The ‘M’ wave pattern shows that both ventricles are not depolarized simultaneously (see animation video in Additional file 3).

The magnitudes of VCG dynamical properties (speed, phase angle, curvature etc.) are mapped into a color scale. Thus, the color coded VCG signals can be plotted in real time to provide extra information besides X, Y, Z, and time scale. As shown in Figure 4, the color coded spatiotemporal VCG representation incorporates extra cardiac vector movement information so as to facilitate doctor’s interpretation of valuable spatiotemporal patterns. Figure 4 displays the speed of each vector movement in a specific color corresponding to the color bar included in the right side of the figure. As illustrated by the color variations in Figure 4 for a patient with the bundle branch block disorder, cardiac vectors in QRS loop move the fastest, T wave the second, P wave the third, and isoelectric points the slowest. It may also be noted that there are two curly R peaks (see Figure 4) which are designated as “*M* waves”, i.e., one of the typical pathological patterns for bundle branch block patients. The “*M* wave” pattern shows that both ventricles are not depolarized simultaneously. The delay in the blocked bundle branch allows the unblocked ventricle to begin depolarizing before the blocked ventricle. This kind of slightly lag effect in one ventricle produces pathological ‘M’ wave and widens the QRS loop.

**Figure 5:**
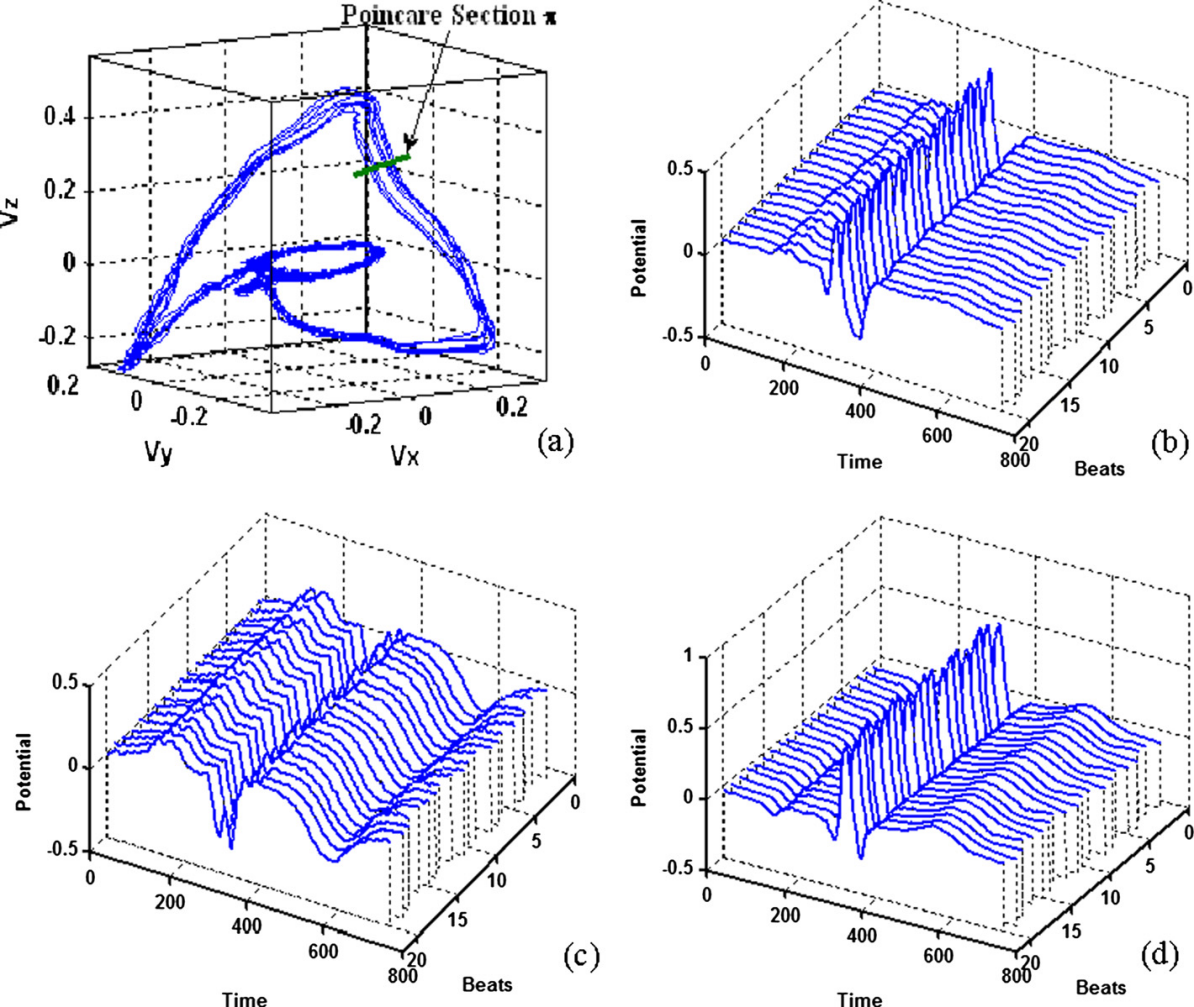
(a) Poincaré sectioning of 3D VCG trajectory; (b) One-dimensional projected cardiac VCG ensembles along X axis; (c) One-dimensional projected cardiac VCG ensembles along Y axis; (d) One-dimensional projected cardiac VCG ensembles along Z axis.

D. ECG Ensemble CharacterizationThe 3D display of cardiac vector loops from several cycles commonly show the near-periodic patterns but with hidden temporal variations between heart cycles. The aligned ECG ensembles will contribute to the gathering of homogeneous majority clusters of ECG signals for selective averaging. The Poincaré section (see Figure 5 (a)) is utilized to characterize the spatiotemporal cardiac patterns in the form of ensembles as well as detailed beat-to-beat variations [18,24]. Here, Poincaré section is a *2-*dimensional hyperplane intersecting with the state space trajectories. The recurrence property of VCG trajectory shows that for every *ϵ* < 0 and almost every cardiac vector $v\left(i\right),\exists j>0$ such that $||v\left(i\right)-v\left(j\right)||<\epsilon$, in effect, the trajectories of VCG vectors remain bounded. Those points at which the trajectory intersects the Poincaré section follow a return map. Figure 5 (b-d) shows the aligned heart beats along the X, Y, Z axes. It may be noted that heart cycles are sharing similar morphologies in any of three orthogonal directions but there exist beat-to-beat variations due to heart rate variability. The time elapsed for the completion of one VCG cycle (P, QRS, T) provides the RR intervals [24]. Heart rate variability will drive some ensembles move faster, i.e., some ECG strands have shorter intervals, compared to the others.

## Results

**Figure 6:**
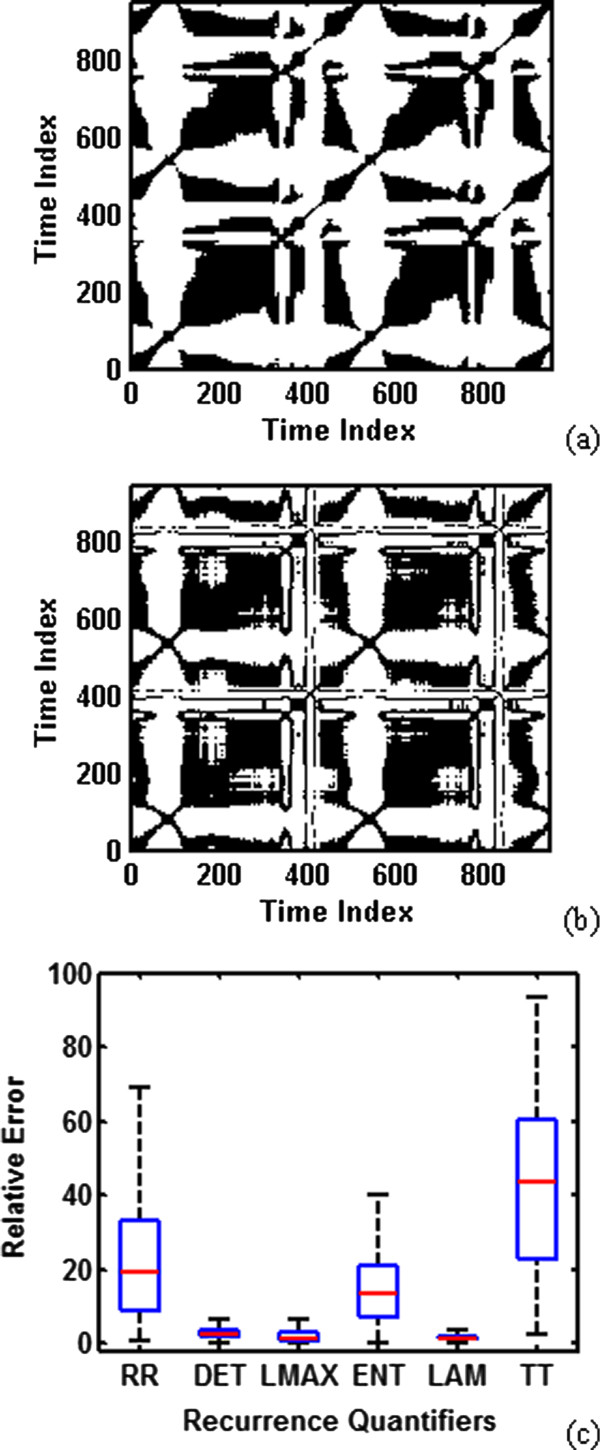
**(a) Recurrence plot of the actual state space using 3-lead VCG signals in patient104/s0306lre. (b)** Recurrence plot of lag-reconstructed state space from lead I ECG signals in patient104/s0306lre. **(c)** Relative errors of recurrence quantifiers between actual and lag-reconstructed state space for 80 health controls.

**Figure 7:**
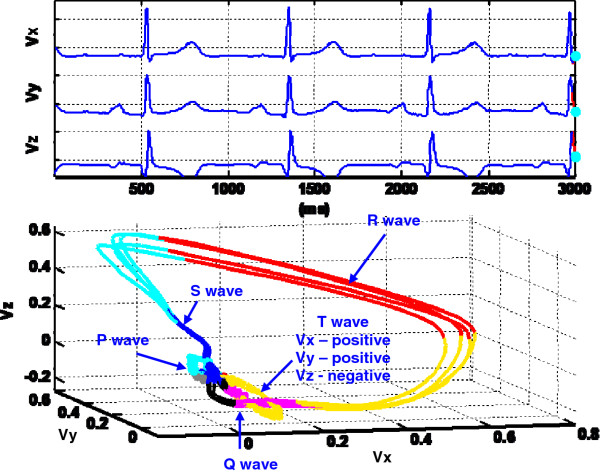
**Color coded dynamic VCG representation plot of a healthy control (HC) subject.** T wave is in yellow color and at the normal position – Octant 6. The appearance of red color in QRS loop indicates the usual position of cardiac electrical axis (see animation video in Additional file 4).

This present investigation is aimed at a new dynamic display of VCG to characterize both spatial and temporal patterns of cardiac electrical activities. The temporal information in the spatiotemporal VCG representation is essential for the identification of cardiac arrhythmia (abnormal heart rhythm such as Bradycardia and Tachycardia). In the 12-lead ECG, Bradycardia is identified as a resting heart rate of <60 beats per minute in the time domain. Similarly, cardiac vectors from Bradycardia patients will rotate for less than 60 cardiac cycles per minute in the dynamic VCG signal representation. The implementation and advantages of dynamic VCG representation are shown as follows:

A. Dynamic VCG Implementation DetailsMost previous investigations utilized the lag-reconstructed ECG representation to quantify nonlinear dynamical patterns, e.g., recurrence statistics and study their correlations with cardiac disorders. The mathematical formulation of recurrence quantifiers is detailed in our previous investigation [25,26] and other references [20,27]. The recurrence plot is defined as: $T_{i,j}:=\Theta \left(\epsilon -||\vec{x}\left(i\right)-\vec{x}\left(j\right)||\right)$, where ϵ is a cutoff distance and **Θ** is the Heaviside function. As shown in Figure 6, the recurrence plot of 3-lead VCG (see Figure 6 a) yields similar recurrence patterns as the lag-reconstructed ECG state space (see Figure 6 b) for the recording of patient104/s0306lre. The black dot indicates that the distance between the states $\vec{x}\left(j\right)$and $\vec{x}\left(j\right)$ is below the cutoff distance ε. The texture patterns in the recurrence plots reveal information of the ECG signal, e.g., the diagonal structures represent the near-periodic patterns. Furthermore, six quantitative features are extracted from recurrence plots to analyze the underlying processes and hidden cardiac rhythms. The recurrence quantifiers include recurrence rate (RR), determinism (DET), maximal length of diagonal structures (LMAX), entropy (ENT), laminarity (LAM) and trapping time (TT) [25-27].The values of recurrence quantifiers are RR:94.63, DET:99.54, LMAX:404, ENT:6.55, LAM:99.64, TT:55.87 for 3-lead VCG (see Figure 6 a), and RR:99.01, DET:97.44, LMAX:410, ENT:5.65, LAM:98.40, TT:37.69 for the lag-reconstructed ECG state space (see Figure 6 b). In addition, we extracted the recurrence quantification statistics for 80 healthy controls and calculated the relative errors. The relative error is calculated as $\left|x_{\mathrm{true}}-x_{\mathrm{pseudo}}\right|/x_{\mathrm{true}})$, where $x_{\mathrm{true}}$ is the quantifier computed from the VCG state space, and $x_{\mathrm{pseudo}}$ is from the lag-reconstructed pseudo state space. The relative errors provide a good measure of how good the recurrence statistics from the lag-reconstructed ECG state space is relative to the 3-lead VCG. As shown in Figure 6 (c), the box plot is used to visualize the distribution of relative errors. The red line in the middle of boxplot represents the median, the blue box shows the lower quartile and upper quartile of data distributions, and the black dash lines represent the most extreme values within 1.5 times the interquartile range. Figure 6 (c) shows that the relative errors of DET, LMAX, and LAM are less than 6.6% for all 80 subjects. The relative errors of RR are less than 18.9% for half of the subjects, and the median relative errors of ENT are less than 13.4%. However, the median relative errors of TT are around 43.2% and higher than the other five recurrence quantifiers. It may be noted that the lag-reconstructed ECG state space approximates the measures of some dynamical properties with small relative errors when the 3-lead VCG is not readily available.Figure 7 shows the color-coded spatiotemporal VCG representation for a healthy control subject. It may be noted that the real-time movements of cardiac vectors can be examined using animations of the dynamic VCG signals (view Additional file 4 for animations enclosed separately). As shown in Figure 7, the peak of T wave loop is in the Octant 6 (XYZ: ++−) with the yellow color. This is true for most of the healthy control cases. Statistical analysis showed that only 6 out of 80 healthy recordings in PTB database are away from Octant 6. In contrast, the peak of T waves from 31.79% (117/368) of MI subjects are found to remain in Octant 6 and the rest (68.21%) spread over all other octants. This indicates that the spatial directions of ventricle repolarization are deviated from octant 6 for the majority (i.e., 68.21%) of MI subjects. Although this is analogous to the T wave inversion patterns in the time domain, it may be noted that a variety of T wave inversions is shown in different leads of the 12-lead ECG. In the spatiotemporal domain, this present investigation revealed that the spatial directions of ventricle repolarization belong to the octant 6 for the majority (i.e., 74/80) of health controls.Therefore, the spatiotemporal VCG representation makes irregular ventricle repolarization behaviors more clear-cut and easier for computer implementation. In addition, the initial portion of QRS loops is ‘Q wave’ when the initial vectors are in opposite direction from the maximal R vector (i.e., scalar product negative), and similarly for terminal vectors as ‘S wave’. A different color scheme can also be added by identifying within the initial 3-D QRS loop with colored dots or other suitable labels 20 ms and 30 ms time points to add temporal information about Q wave duration. The Q wave and S wave can be located by combining the temporal XYZ display in the top and the real time motion of cardiac vectors in the bottom of spatiotemporal VCG representation.

**Figure 8:**
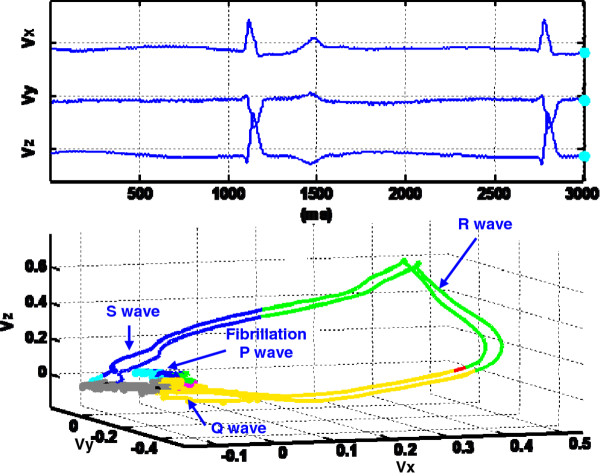
**Color coded dynamic VCG representation plot of a patient with dysrhythmia and atrial fibrillation.** The irregular P wave morphology shows abnormal excitations from SA node to Atrial. The disappearance of red and appearance of green color in QRS loop also indicate abnormal locations of cardiac electrical axis (see animation video in Additional file 5).

B. Benefits of Dynamic VCG representationThe dynamic VCG representation further enhances our previous investigations [18,25] to characterize the spatiotemporal VCG patterns and automatically assess cardiovascular conditions. We showed that recurrence quantifiers extracted from 3-lead VCG are good indicators of myocardial infarction [18,25]. Dynamic VCG representation possesses advantages to disclose space-time pathological characteristics unknown, or not feasible, from the time-domain ECG signals. Figure 8 shows color coded spatiotemporal VCG representation for a dysrhythmia and atrial fibrillation patient. Atrial fibrillation [28], due to continuous rapid-firing of many atrial automaticity foci, typically produces tiny and wavy ECG spikes instead of identifiable P waves. In the dynamic VCG representation, P loops is not clearly distinguishable and the VCG trajectory appears to be chaotic before entering the QRS loops. Such erratic cardiac vector movements during atrial electrical activity closely correlate with the rapid-firing of multiple irritable atrial foci. Multi-dimensional spatial view provides a complete picture of abnormal atrial electrical activities and facilitates the computer based multivariate analysis. If the spatiotemporal cardiac electrical activity is projected along the direction of V1-V2 leads, it is easier to visualize the prominent fibrillation waves in the time domain. However, the projection of spatiotemporal cardiac electrical activity diminishes important multi-dimensional information of cardiac pathological behaviors into one dimension. As such, there is an information loss in spite of the fact of easy visualization. In other words, if a spatial vector is projected into an axis that does not contain this vector, there will be information loss.It may also be noted in Figure 8 that the RR interval is longer than the normal case mainly because of the long time taken for the excitation of atrium (isopotential duration). In addition, the color changes of the VCG trajectory with respect to octant numbers are vastly different from the normal case shown in Figure 7. The disappearance of red and the appearance of green color instead in the QRS loop also indicate the abnormal locations of cardiac electrical axis, which are pathological patterns for some diseases. The cardiac electrical axis refers to the depolarization direction of ventricular myocardium, which is conventionally defined based on the QRS complexes of leads I, II, III, AVL, AVF, AVR in the frontal plane and V1-V6 in the horizontal plane. This is similar to determine the direction of ventricular repolarization in 3D space. Therefore, the distribution of the QRS loop along various octants, i.e., colors of the QRS loop, provides an analogous measure of the heart electrical axis.As shown in Figures 4, 7 and 8, pathological patterns of cardiac disorders were characterized from the spatiotemporal VCG representation. Spatiotemporal VCG signal representation enhances the automatic assessment of cardiovascular diseases. The software color-coding scheme facilitates in the quantification of space-time cardiac pathological patterns.

## Conclusions

In this investigation, we have made an attempt to capture the spatiotemporal characteristics of the VCG signals by viewing the cardiac vectors in real time on a computer monitor screen instead of a static signal output on a paper which at best can record 3 dimensions. This approach overcomes the drawbacks of conventional static VCG representation and provides concurrent spatial and temporal resolutions. The alternative lag-reconstructed approach from nonlinear dynamics principles addresses the sometime difficult situations for the study of cardiac state space when there is only 1-lead ECG signal available. The spatiotemporal VCG representation incorporates additional dynamical properties of cardiac vector movements (curvature, velocity, octant, and phase angle etc.) with the color coding scheme. Furthermore, Poincaré sectioning of the 3D VCG vector loops extracts the homogeneous ECG ensembles for the information on cardiac cycle-to-cycle self-similarities and variations. It is shown that the proposed dynamic VCG approach surmounts some drawbacks of both 12-lead ECG and static VCG representation, and provides critical spatial as well as temporal information of the heart dynamics. The cardiovascular pathological patterns are found to be effectively captured by this new 3D dynamic representation approach. The presence of both spatial and temporal characteristics in dynamic representation improves the automatic assessment of cardiovascular diseases with the use of VCG signals.

## Competing interests

The authors declare that they have no competing interests.

## Authors’ contribution

HY contributed to the development of spatiotemporal methods and tools, evaluated the data, performed the data analysis, and drafted the manuscript as part of his PhD work under the advisement of the other two co-authors (SB and RK). SB contributed to the design of the study and revised the manuscript. RK conceived the study and revised the manuscript. All authors read and approved the final manuscript.

## Dedication

This paper is respectfully dedicated to the memory of Dr. Ranga Komanduri (1942-2011) who was the originator of this manuscript.

## Supplementary Material

Additional file 1 ** Animation video for the spatiotemporal VCG representation of a patient with myocardial infarction, anterior.** The top plot displays VCG signals in three vector components as a function of time, and the bottom part shows the real time cardiac vector movement in the 3D space. Head (green) gives the current position of cardiac vector. Tail (red) indicates the direction and rotation of cardiac vector movements. Click here for file

Additional file 2 ** Animation video for Lag-reconstructed dynamic ECG representation for a normal subject.** Time delay coordinates of the original one dimensional ECG time series are used to embed the 3D manifold. Click here for file

Additional file 3 ** Animation video for dynamic VCG representation of a patient with bundle branch block.** The 3D VCG trajectory is color coded with the potential variation rate listed in the right side. Two curly R peaks designated as “M waves” are shown in the QRS loop. The ‘M’ wave pattern shows that both ventricles are not depolarized simultaneously. Click here for file

Additional file 4 ** Animation video for color coded dynamic VCG representation of a healthy control (HC) subject. T wave is in yellow color and at the normal position – Octant 6.** The appearance of red color in QRS loop indicates the usual position of cardiac electrical axis. Click here for file

Additional file 5 ** Animation video for Color coded dynamic VCG representation of a patient with dysrhythmia and atrial fibrillation.** The irregular P wave morphology shows abnormal excitations from SA node to Atrial. The disappearance of red and appearance of green color in QRS loop also indicate abnormal locations of cardiac electrical axis. Click here for file
